# *Faecalibacterium prausnitzii* prevents physiological damages in a chronic low-grade inflammation murine model

**DOI:** 10.1186/s12866-015-0400-1

**Published:** 2015-03-21

**Authors:** Rebeca Martín, Sylvie Miquel, Florian Chain, Jane M Natividad, Jennifer Jury, Jun Lu, Harry Sokol, Vassilia Theodorou, Premysl Bercik, Elena F Verdu, Philippe Langella, Luis G Bermúdez-Humarán

**Affiliations:** INRA, Commensal and Probiotics-Host Interactions Laboratory, UMR 1319 Micalis, F-78350 Jouy-en-Josas, France; AgroParisTech, UMR1319 Micalis, F-78350 Jouy-en-Josas, France; Farncombe Family Digestive Health Research Institute, McMaster University, 1200 Main St West, H.Sc. 3N6, Hamilton, Ontario Canada; INSERM, Equipe AVENIR U1057 / UMR CNRS 7203, 75012 Paris, France; Department of Gastroenterology and Nutrition, AP-HP, Hôpital Saint-Antoine F-75012 and UPMC Univ Paris 06F-75005, Paris, France; INRA, Neuro-Gastroenterology and Nutrition Team, UMR 1331 Toxalim, F-31931 Toulouse, France

**Keywords:** Microbiota, Dysbiosis, IBD-remission, Low-grade inflammation, Probiotics

## Abstract

**Background:**

The human gut houses one of the most complex and abundant ecosystems composed of up to 10^13^-10^14^ microorganisms. The importance of this intestinal microbiota is highlighted when a disruption of the intestinal ecosystem equilibrium appears (a phenomenon called dysbiosis) leading to an illness status, such as inflammatory bowel diseases (IBD). Indeed, the reduction of the commensal bacterium *Faecalibacterium prausnitzii* (one of the most prevalent intestinal bacterial species in healthy adults) has been correlated with several diseases, including IBD, and most importantly, it has been shown that this bacterium has anti-inflammatory and protective effects in pre-clinical models of colitis. Some dysbiosis disorders are characterized by functional and physiological alterations. Here, we report the beneficial effects of *F. prausnitzii* in the physiological changes induced by a chronic low-grade inflammation in a murine model. Chronic low-grade inflammation and gut dysfunction were induced in mice by two episodes of dinitro-benzene sulfonic acid (DNBS) instillations. Markers of inflammation, gut permeability, colonic serotonin and cytokine levels were studied. The effects of *F. prausnitzii* strain A2-165 and its culture supernatant (SN) were then investigated.

**Results:**

No significant differences were observed in classical inflammation markers confirming that inflammation was subclinical. However, gut permeability, colonic serotonin levels and the colonic levels of the cytokines IL-6, INF-γ, IL-4 and IL-22 were higher in DNBS-treated than in untreated mice. Importantly, mice treated with either *F. prausnitzii* or its SN exhibited significant decreases in intestinal permeability, tissue cytokines and serotonin levels.

**Conclusions:**

Our results show that *F. prausnitzii* and its SN had beneficial effects on intestinal epithelial barrier impairment in a chronic low-grade inflammation model. These observations confirm the potential of this bacterium as a novel probiotic treatment in the management of gut dysfunction and low-grade inflammation.

**Electronic supplementary material:**

The online version of this article (doi:10.1186/s12866-015-0400-1) contains supplementary material, which is available to authorized users.

## Background

The human gastrointestinal tract (GIT) is one of the most complex ecosystems. The interaction between GIT microbiota and the host is indispensable for the maturation of the immune system and for the regulation of intestinal physiology [1]. Firmicutes (which encompasses *Clostridum leptum* and *C. coccoides*) and Bacteroidetes are the two dominant phyla in the human gut, representing 90% of the microbiota, with Actinobacteria being the third, comprising of only 3% [2].

When the normal microbial ecosystem is disrupted, different non-predominant bacteria can bloom, constituting a potential trigger for disease. The term dysbiosis refers to a type of microbial imbalance that may promote disease. The strongest relationship between dysbiosis and pathological condition has been observed in inflammatory bowel diseases (IBD), where the proportion of Firmicutes is lower than in healthy subjects [3,4]. IBD, composed mainly of ulcerative colitis (UC) and Crohn’s disease (CD), are characterized by an unusual activation of the GIT-immune system leading to a chronic inflammation of the gut with a mix of remission and exacerbation periods. It has recently been suggested that the colonic microbiota is also abnormal in patients with irritable bowel syndrome (IBS), with a 2-fold increased ratio of the Firmicutes to Bacteroidetes [5]. IBS is a symptom-based diagnosis characterized by chronic abdominal pain and altered bowel habits [6] in the absence of detectable structural abnormalities [7].

IBD and IBS are classically considered as dichotomous conditions. However, increasing evidence, such as the involvement of brain-gut axis dysfunction [8-10], as well as abnormal microbiota, supports some pathogenetic overlap between the two conditions [8]. Furthermore, the identification of low-grade inflammation [9], histopathological observations and mucosal cytokine levels in the colon of a subset of IBS patients [10,11] and the presence of IBS-like symptoms in quiescent IBD [8,12-14] support this hypothesis.

Low-grade inflammation can persist in many IBD patients who have otherwise obtained clinical remission [15], as well as barrier alterations due to functional impairment of tight junction proteins [16]. In fact, impaired gut barrier function appears to precede the development of IBD [17] and increased epithelial permeability causes mucosal immune activation predisposing or enhancing disease progression [18].

Due to the importance of the microbiota in the development of these dysbiosis-related diseases, there is currently increasing interest in the development of microbiota-modulating therapies, including the use of probiotics [19,20]. *F. prausnitzii* is a candidate probiotic bacterium that has been proposed as a sensor of intestinal health [21]. Changes in the abundance of this commensal anti-inflammatory bacterium [22] and major member of the *Clostridium leptum* group [23] have been linked to dysbiosis in several human disorders [21], such as IBD, where a low abundance was found in patients exhibiting endoscopic recurrence 6 months after surgery [3]. Furthermore, the recovery of *F. prausnitzii* counts after relapse is associated with maintenance of clinical remission in UC patients [24]. Besides, *F. prausnitzii* has shown protective effects in inflammation parameters in both acute [22] and chronic [25] models of chemical-induced active inflammation; however its beneficial role during low-grade inflammation and gut dysfunction has not been tested yet. For these reasons, we hypothesized a possible protective role of *F. pausnitzii* on gut dysfunction mediated by low-grade inflammation. Therefore, we investigated the potential of *F. prausnitzii* as a probiotic for gut dysfunction prevention and therapy. We tested the effects of *F. prausnitzii* in a murine model of chronic low-grade inflammation mimicking some of the most common gut dysfunction parameters found in IBD patients under remission.

## Results

### Validation of a low-grade inflammation model in DNBS-treated mice

**Figure 1:**
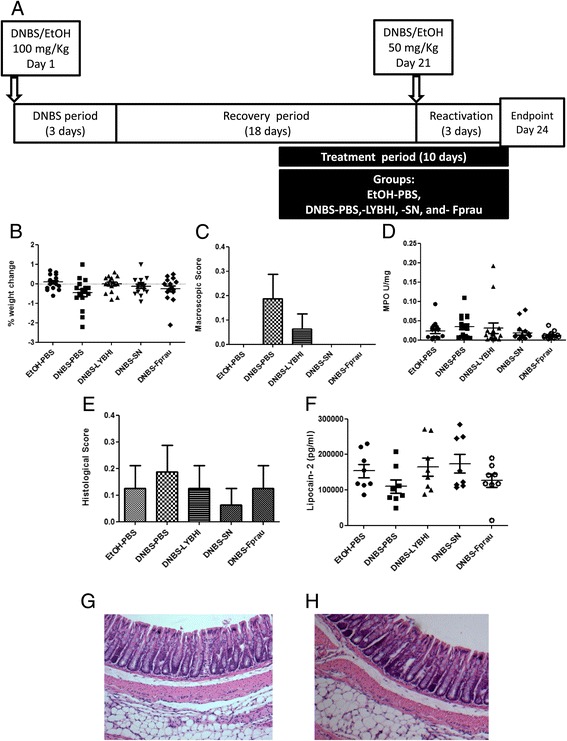
**Experimental protocols for the mouse model of chronic micro-inflammation and absence of inflammation. A)** Colitis was induced by intrarectal administration of 100 mg/Kg of DNBS in solution in 30% ethanol (EtOH). Control mice (without colitis) received only 30% EtOH. The effects of DNBS are highest during the first 3 days after its administration (DNBS period). Ten days after the end of the DNBS period, bacterial culture, PBS, or supernatant (SN) were intragastrically administered daily for 10 days (gavage period). Colitis was reactivated 21 days after the first DNBS injection with a second injection of 50 mg/Kg of DNBS solution. Three days after reactivation mice were sacrificed. The severity of the reactivated colitis was assessed from difference in body weight **(B)**, macroscopic score **(C)**, MPO activity **(D)**, histological score **(E)** (*n =* 16 mice per group) and lipocalin-2 levels **(F)** (n = 2*4 = 8 mice per group) between control non-inflamed (EtOH-PBS), control inflamed (DNBS-PBS), bacteria-free culture medium (DNBS-LYBHI), *F. prausnitzii* strain A2-165 (DNBS-Fprau), and *F. prausnitzii* A2-165 SN (DNBS-SN) groups. Experiments were performed at least in duplicate. Normal appearance of the colon of a control mouse with no inflammation **(G)** and of a mouse with micro-inflammation **(H)**.

**Figure 2:**
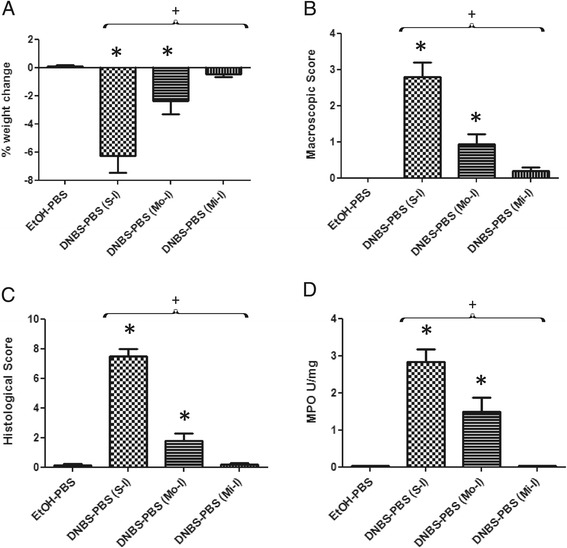
**Comparison of inflammation measures in severe and moderate colitis models** **[** 25 **]** **with the micro-inflammation model employed in this study.** Severity of the colitis was assessed from differences in body weight **(A)**, macroscopic score **(B)**, histological score **(C)** and MPO activity **(D)** between the control non-inflamed (EtOH-PBS), control inflamed severe colitis model (DNBS-PBS (S-I)) [25], moderate colitis model (DNBS-PBS (M-I)) [25] and micro-inflammation model (DNBS-PBS (Mi-I)). Experiments were performed at least in duplicate. Comparisons involved the non-parametric Kruskal-Wallis test was used followed by a Dunn’s Multiple Comparison test. *p < 0.05 *vs.* DNBS-PBS, +*p <* 0.05 (*n =* 16 mice per group).

The model of low-grade DNBS inflammation developed in this study involves the induction of a low grade, subclinical inflammatory status, followed by a recovery period and a reactivation period mimicking the relapsing nature in IBD (Figure 1A). The challenge with a sub-colitic DNBS dose (i.e. second DNBS administration) did not induce important changes in weight or colonic macroscopic or histological scores confirming the absence of a strong inflammation (Figure 1B, C, E, G, H). The degree of infiltration by polymorphonuclear neutrophils, as measured by tissue MPO activity, as well as Lipocalin-2 (Lcn-2), an early inflammation marker in inflammatory and autoimmune disorders, did not differ significantly between DNBS-treated and control animals (Figure 1D, F). These observations suggest that DNBS-treated mice tend to display a low-grade inflammatory status rather than a strong inflammation (as observed in IBD patients) thereby validating our model for further analysis of physiological changes induced by this chronic low-grade, subclinical inflammation. The findings for all these markers thus suggest that the colons of DNBS-treated mice have a low-grade inflammatory status. Comparison of the levels of these markers with those previously published by our group following a colitic dose of DNBS that induced severe or moderate chronic inflammation [25] provided further evidence that this protocol leads to low-grade inflammatory conditions (Figure 2).

### *Faecalibacterium prausnitzii* A2-165 and its SN counteract DNBS-induced hyperpermeability *in vivo*

**Figure 3:**
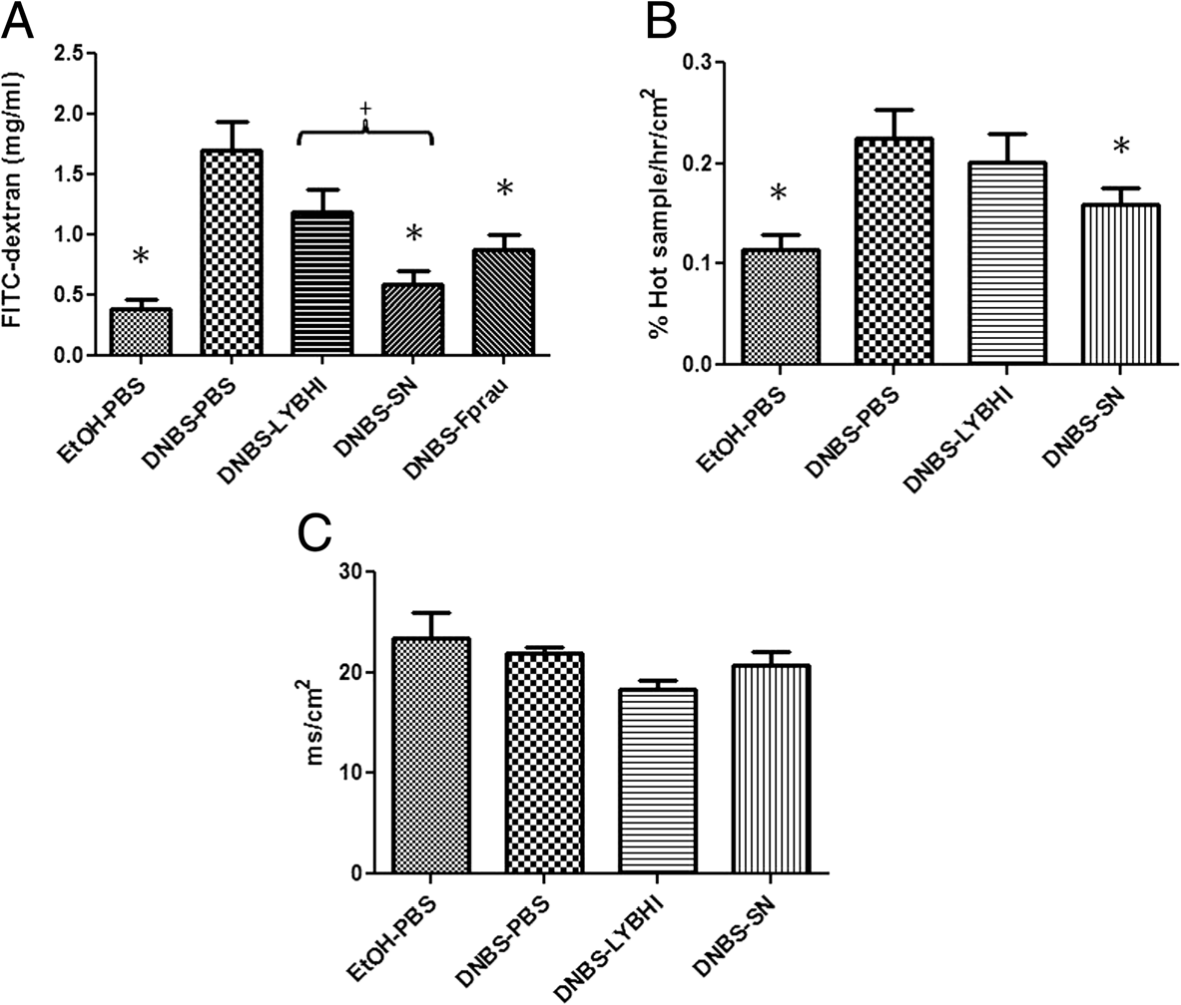
***In vivo*** **and** ***in vitro*** **permeability measurements.** For *in vivo* measurements of gut permeability, animals were orally gavaged with FITC-dextran **(A)** (*n =* 16 mice per group). For *in vitro* measures, sections of colon were mounted in Ussing chambers and ^51^Cr-EDTA flux (% Hot Sample/hr/cm^2^) **(B)** and tissue conductance (mS/cm^2^) **(C)** were measured (*n =* 6 mice per group). Control non-inflamed (EtOH-PBS), control inflamed (DNBS-PBS), bacteria-free culture medium (DNBS-LYBHI), *F. prausnitzii* strain A2-165 (DNBS-Fprau), and *F. prausnitzii* A2-165 SN (DNBS-SN) groups. Experiments were performed at least in duplicate. Comparisons involved the non-parametric Kruskal-Wallis test was used followed by a Dunn’s Multiple Comparison test. *p < 0.05 *vs.* DNBS-PBS, +*p <* 0.05.

The gut of DNBS-treated mice showed high permeability to the paracellular tracer FITC-dextran *in vivo* (Kruskal-Wallis test followed by a Dunn’s Multiple Comparison test, *p <* 0.05) (Figure 3A). We tested whether *F. prausnitzii* modulates this DNBS-induced gut dysfunction. Treatment with either *F. prausnitzii* or its SN resulted in a significant reduction of intestinal permeability (Kruskal-Wallis test followed by a Dunn’s Multiple Comparison test, *p <* 0.05) (Figure 3A). To confirm that these findings corresponded to local paracellular translocation (*i.e.* in the colon) of the FITC-dextran *in vivo* in this model, intestinal permeability was assessed *in vitro* in colonic samples mounted in Ussing chambers extracted from mice at sacrifice (Figure 3B). The results were consistent with the pattern observed *in vivo* (Kruskal-Wallis test followed by a Dunn’s Multiple Comparison test, *p <* 0.05) confirming local translocation of the FITC-dextran. We then tested *F. prausnitzii* SN (which had a large effect on intestinal permeability *in vivo*) with samples from DNBS-challenged mice by Ussing chamber analysis (Figure 3B): the ^51^Cr-EDTA flux, measured in % Hot Sample/hr/cm^2^, was lower with SN than PBS (Kruskal-Wallis test followed by a Dunn's Multiple Comparison test, *p* < 0.05) (Figure 3B). No significant differences were obtained in tissue conductance (mS/cm^2^) (Figure 3C).

### *F. prausnitzii* restores DNBS-induced apical junction proteins decrease

**Figure 4:**
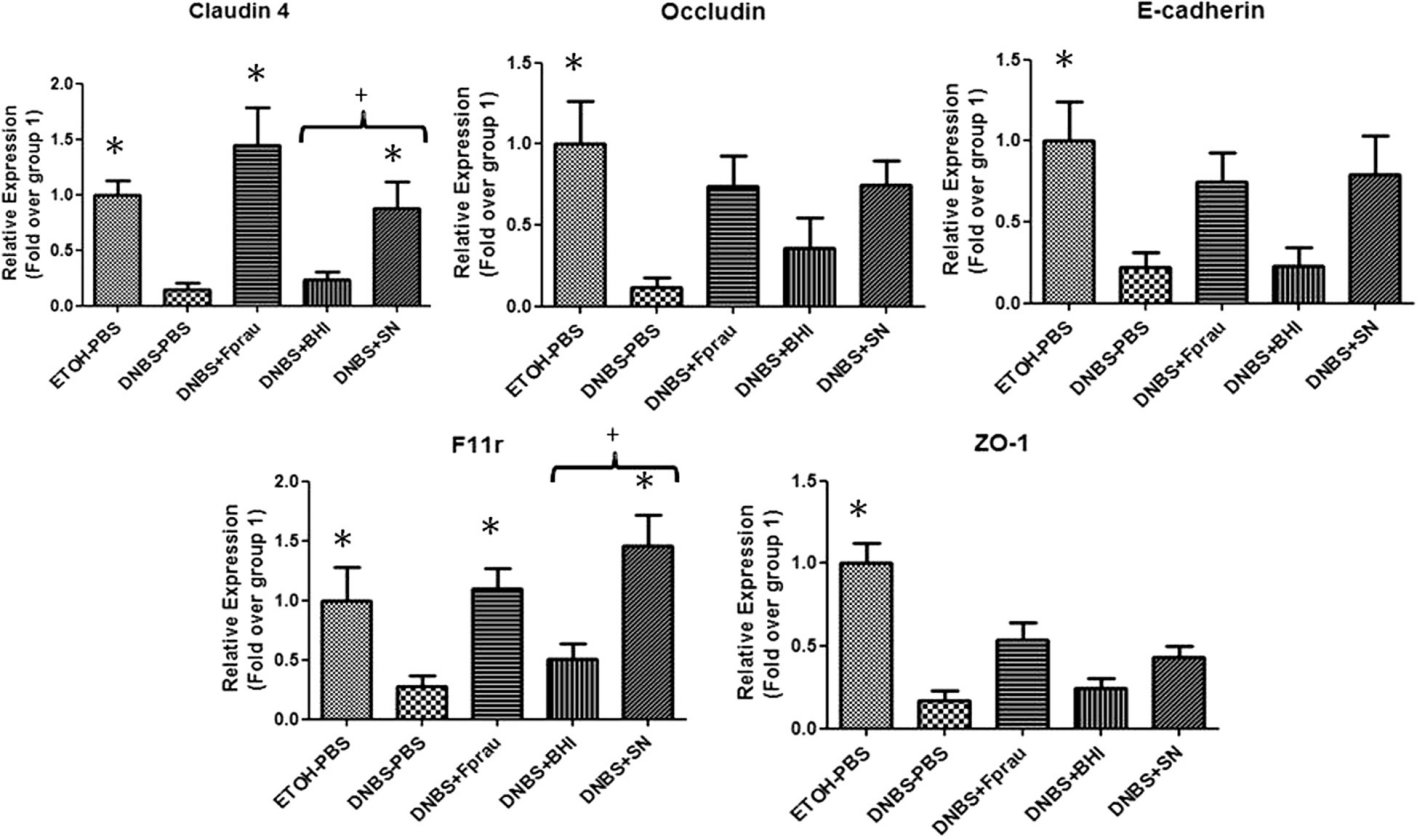
**Effect of** ***F. prausnitzii*** **on apical junction proteins mRNAs in a DNBS-induced low-grade inflammation model.** Apical junction protein expression levels were determined by real-time qPCR. Control non-inflamed (EtOH-PBS), control inflamed (DNBS-PBS), bacteria-free culture medium (DNBS-LYBHI), *F. prausnitzii* strain A2-165 (DNBS-Fprau), and *F. prausnitzii* A2-165 SN (DNBS-SN) groups. Experiments were performed at least in duplicate. Comparisons involved the non-parametric Kruskal-Wallis test was used followed by a Dunn’s Multiple Comparison test. *p < 0.05 *vs.* DNBS-PBS, +*p <* 0.05 (*n =* 6 mice per group).

These effects on gut permeability prompted us to investigate the involvement of epithelial adherens junction (AJ) and tight junction (TJ) proteins, by assaying for relevant mRNA levels. The mRNA levels for Claudin-4 (Kruskal-Wallis test ,*p* = 0.0006), E-cadherin (Kruskal-Wallis test, *p* = 0.0175), F11r (JAM1) (Kruskal-Wallis test, *p* = 0.0023), Occludin (Kruskal-Wallis test, *p* = 0.0170) and ZO-1 (Kruskal-Wallis test, *p* = 0.0001) were all less abundant in DNBS-treated mice than in control mice (Kruskal-Wallis test followed by a Dunn’s Multiple Comparison test, *p <* 0.05) (Figure 4); Claudin-1, 2, 5 and 15 mRNA levels were unaffected (data no shown). *F. prausnitzii* and its SN tended to normalize the amounts of all these mRNA transcripts, the only statistically significant regulation was found for Claudin-4 and F11r (Kruskal-Wallis test followed by a Dunn’s Multiple Comparison test, *p <* 0.05) (Figure 4). Claudin-4, E-cadherin, F11r and Occludin expression was also analyzed by immunofluorescence (Additional file 1: Figure S1). Staining for the four proteins was reduced in DNBS + PBS mice compared to EtOH + PBS group (Additional file 1: Figure S1). According to the qPCR results, *F. prausnitzii* tends to increase the expression of all the proteins.

### A systemic and mucosal reduction in pro-inflammatory cytokine production correlates with the protective effects of *F. prausnitzii* and its SN after DNBS challenge

**Figure 5:**
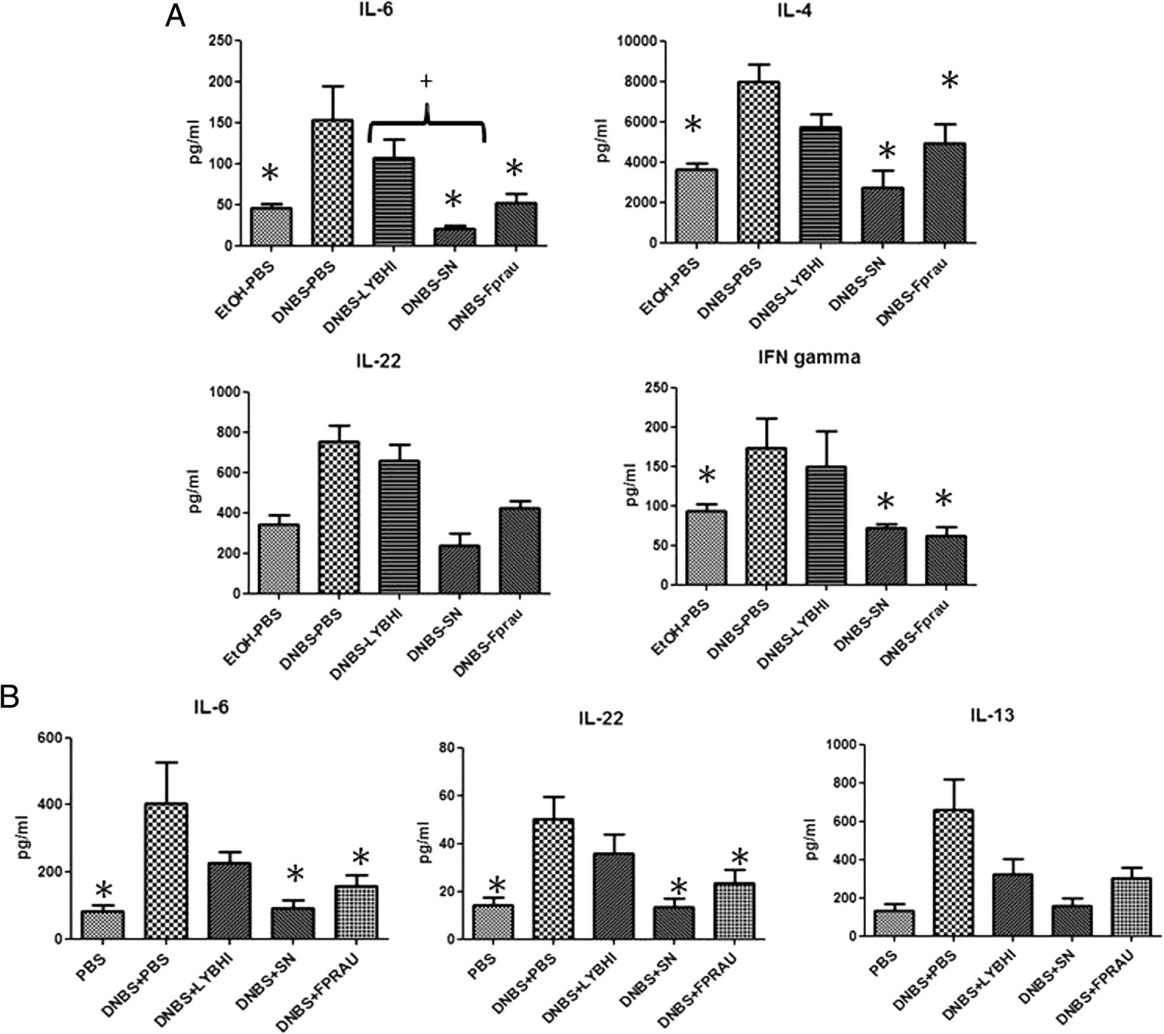
**Cytokine concentrations in colon (A) and serum (B) in the DNBS micro-inflammation model.** Control non-inflamed (EtOH-PBS), control inflamed (DNBS-PBS), bacteria-free culture medium (DNBS-LYBHI), *F. prausnitzii* strain A2-165 (DNBS-Fprau), and *F. prausnitzii* A2-165 SN (DNBS-SN) groups. Experiments were performed at least in duplicate. Comparisons involved the non-parametric Kruskal-Wallis test was used followed by a Dunn’s Multiple Comparison test. *p < 0.05 *vs.* DNBS-PBS, +*p <* 0.05 (*n =* 16 mice per group).

We investigated the cytokines involved in this low-grade inflammation model and the mechanism by which *F. prausnitzii* and its SN protect the intestinal mucosa. We assayed 13 cytokines (including those involved in the Th1, Th2, Th17 and Th22 pathways) in both colonic and serum samples after the second DNBS injection (Figure 5). The concentrations of IL-13, IL-1α, IL-6, IL-22, IL-2, IL-27, IL-4, IFN-γ, and TNF-α in colon samples were higher in DNBS-treated mice than in controls (Figure 5A and data not shown), consistent with local inflammation. Similarly, cytokine concentrations in serum samples were consistent with the low-grade inflammatory status (Figure 5B and data not shown). Strikingly, the concentration of IL-6, IFN-γ and IL-4 in colon samples and of IL-6 and IL-22 in serum was statistically significantly reduced by treatment with either *F. prausnitzii* or its SN (Kruskal-Wallis test followed by a Dunn’s Multiple Comparison test, *p <* 0.05) and there were clear tendencies for these treatments to reduce colonic IL-22 and serum IL-13 concentrations (Figure 5).

### *F. prausnitzii* and its SN restore serotonin variations observed in DNBS-treated mice

**Figure 6:**
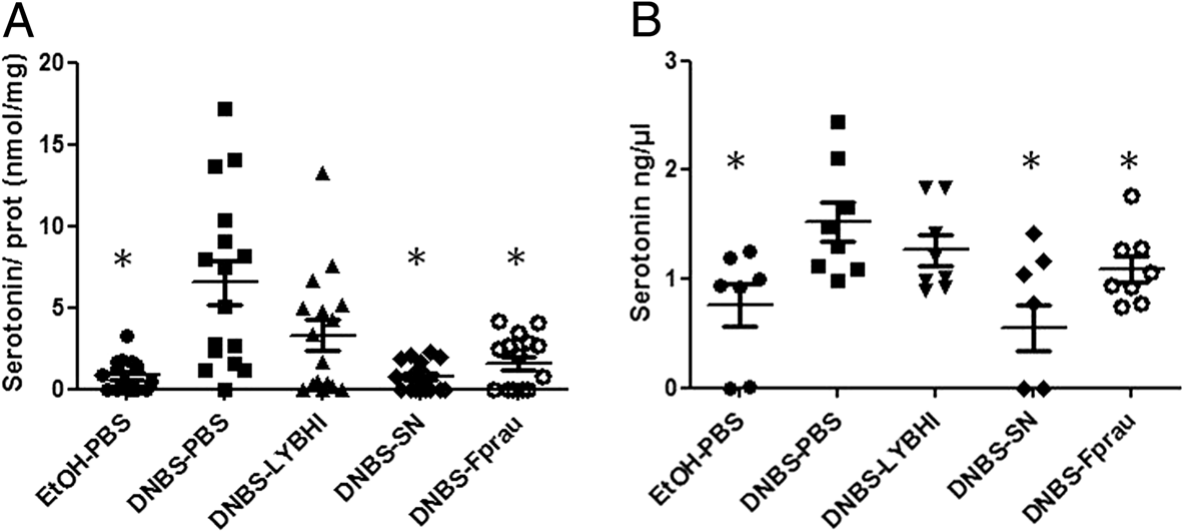
**Serotonin concentrations in colon (A) and colon contents (B) in the DNBS micro-inflammation model.** Control non-inflamed (EtOH-PBS), control inflamed (DNBS-PBS), bacteria-free culture medium (DNBS-LYBHI), *F. prausnitzii* strain A2-165 (DNBS-Fprau), and *F. prausnitzii* A2-165 SN (DNBS-SN) groups. Experiments were performed at least in duplicate.Comparisons involved the non-parametric Kruskal-Wallis test was used followed by a Dunn’s Multiple Comparison test. *p < 0.05 *vs.* DNBS-PBS, +*p <* 0.05 (*n =* 16 mice per group for analysis of colon samples and *n =* 8 mice per group for analysis of colon content samples).

Serotonin (also called 5-HT for 5-hydroxyptamine) of the key neurotransmitters in the GIT, and affects motility, sensation and secretion [26]. We therefore studied serotonin concentrations in colon tissue and content by ELISA. Both colonic (Figure 6A) and colon content (Figure 6B) serotonin concentrations were higher in DNBS-treated mice than EtOH-treated controls (Kruskal-Wallis test followed by a Dunn’s Multiple Comparison test, *p <* 0.05). Treatments with both *F. prausnitzii* and its SN counteracted these increases (Kruskal-Wallis test followed by a Dunn’s Multiple Comparison test, *p <* 0.05) (Figure 6A, B). Serotonin reuptake transporter (SERT) mRNA was assayed by qPCR in colon tissues: the value for inflamed mice was half than for control non-inflamed mice (2^-ΔΔCt^ = 0.49); the value for mice treated with *F. prausnitzii* was the same as that for normal healthy mice (2^-ΔΔCt^ = 1.09 *vs.* EtOH-PBS). Similar positive effects were found in SN-treated mice (2^-ΔΔCt^ = 1.29 *vs.* EtOH-PBS) although the culture medium (LYBHI) itself had a positive effect (2^-ΔΔCt^ = 0.98 *vs.* EtOH-PBS).

## Discussion

There is growing evidence pointing at the role of the gut microbiota in the development of IBD [3,4]. Furthermore, at least 40% of IBD patients in clinical remission have ongoing histological evidence of inflammation [15] and suffer from altered gut functions with the involvement of brain-gut axis dysfunction [8,26,27]. Interestingly, previous transient or chronic inflammation can lead to persistent gut dysfunction [28] and it has been suggested that the host microbiota may affect the two-ways communication pathway between the gut and the brain [29].

Because the microbiota is a determinant of normal gut function and immunity [30], and because there is evidence of dysbiosis in IBD, there is increasing interest in the development of new adjuvant therapies based on modulation of the microbiota [31]. The traditional probiotic approach based on lactic acid bacteria (LAB) strains can be now extended to new candidate beneficial bacteria from the intestinal microbiota, including *F. prausnitzii*. This metabolic active species is a member of the *C. leptum* group, and is one of the most prevalent commensal bacteria in the human gut [2], involved in colonic epithelial physiology [32], and a reduction in its abundance has been observed in IBD patients [24]. The anti-inflammatory properties of the *F. prausnitzii* reference strain A2-165 have been demonstrated *in vitro* in various cellular models and validated *in vivo* in murine models of TNBS-induced acute colitis [22] and DNBS-induced chronic severe and moderate colitis [25].

We investigated the beneficial effects of both *F. prausnitzii* and its filtered SN (with the consequent loss of high molecular weight molecules) using a chronic low-grade inflammatory model, which mimics gut dysfunction related to colonic low-grade inflammation [11,12]. We adjusted the dose of DNBS such that the challenge induced low-grade inflammation, as it has been described for TNBS [33], a model that resembles DNBS [34]. To mimic the relapsing character of IBD and reach a remission status, we used a chronic protocol with recovery and reactivation periods. Our results show that chronic, low-dose administration of DNBS did not induce severe colonic inflammation: there was no macro- or microscopic damages or granulocyte infiltration. Furthermore, no increase in the levels of lipocalin 2 (Lcn-2), an early marker of inflammation, was observed [35]. Lcn-2 is an innate immune protein produced by a variety of cells, and its levels are increased during several pathologic conditions, including immune complex-mediated inflammatory or autoimmune disorders [36,37]. These various results indicate the absence of any substantial active inflammatory tone in this DNBS-treated mouse model. However, the presence of a low-grade inflammation was confirmed by the small increases in some pro-inflammatory cytokine titers (including IL-6 and INF-γ). The levels of most of the cytokines investigated, mainly in serum, were low, with no statistically significant difference between groups. Nevertheless, serum IL-13, colonic IFN-γ and IL-4, and both serum and colonic IL-6 and IL-22 were higher in DNBS treated mice. IFN-γ is a Th1 cytokine and an important activator of macrophages; it is involved in the regulation of inflammation and in barrier dysfunction through the redistribution and expression of TJ proteins [38]. The pro-inflammatory Th1 cytokine IL-6 modulates the excitability of submucosal neurons and increases colonic secretory function. The cytokine IL-22 regulates various processes linked to several autoimmune disorders [39] exerting both antimicrobial and pro-inflammatory functions. IL-4 is a Th-2 cytokine, canonical of IL-13 [40], with stimulatory effects on B- and T-cells [41]; as well as involvement in dichotomous regulation of Th cell differentiation, IL-4 induces so-called alternative macrophage activation [42]. IL-4 and IL-13 can decrease cellular trans-epithelial electric resistance (TEER) *in vitro* and *in vivo* [43,44]. Our findings for the cytokines are difficult to interpret as they seem to involve both Th-1 and Th-2 immune responses as well as effects in the Th-17/Th-22 subset. This is not surprising because the particular inflammatory environment of our model could be the consequence of cross-regulation between all these possible pathways [11]. Although the profile of cytokines after chronic low dose DNBS challenge is not completely characterized, the altered cytokine profiles we document here in DNBS-treated mice and untreated control mice confirm mild immunological dysfunctions in the model. Furthermore, both *F. prausnitzii* and SN treatments had beneficial effects on indicators of immune status.

As IBD patients in remission often exhibit gut dysfunction [45], we therefore chose to study colonic permeability and serotonin levels as functional markers in our model. Treatment with either *F. prausnitzzi* or its SN normalized *in vivo* permeability, suggesting that overall permeability was improved. SN also improved permeability *in vitro* suggesting that the SN acts to protect the paracellular pathway in DNBS-challenged mice. This result is consistent with the findings by Carlssonn *et al.* that *F. prausnitzii* SN counteracted the negative effects of DSS challenge on colonic permeability [46]. Increased permeability after reactivation was accompanied by a reduction in the expression of apical junction proteins. Treatment with either *F. prausnitzii* or its SN restored the levels of Claudin-4 and the junctional adhesion molecule F11r (JAM1). Overall, our results suggest that the protective effect of *F. prausnitzii* and its SN may be the consequence, at least in part, of an effect on tight junctions mediated through certain tight junction proteins. Other common biomarkers in gut dysfunction are related to gut dysmotility [47]. Although serotonin is not a direct marker of motility, it stimulates peristalsis, secretion, vasodilatation and sensory signaling in the gut [48]. It also activates the immune cells to produce pro-inflammatory mediators [49] being a link between inflammation and functional symptoms. Enterochromaffin cells (EC) containing serotonin transduce chemical and mechanical stimuli from the intestinal lumen by releasing the serotonin [50]. Serotonin selective reuptake transporter (SERT), expressed on intestinal enterocytes, terminates the action of 5-HT by eliminating it from the interstitial space [48]. Dysfunctional mucosal serotonin signaling has been implicated in visceral sensitivity and altered motility in IBD patients [50,51]. Colonic levels of 5-HT were high in DNBS-challenged mice, both in colon tissue and content. This result is in agreement with previous studies reporting altered levels of this hormone in murine models of TNBS-induced inflammation and low grade inflammation [33]. Both *F. prausnitzii* and its SN were able to restore the serotonin level to normal. In T-cell-mediated models of inflammation, an increase in EC and a decrease SERT function have been described [52]. The cytokines IFN-γ and IL-13 (restored in both *F. prausnitzii*- and SN-treated mice) are involved in the metabolism of 5-HT, IFN-γ decreases SERT expression in human epithelial Caco-2 cells *in vitro* [53], and IL-13 contributes to increasing EC numbers [52]. In agreement with what has been described for TNBS [54], we found lower SERT levels in DNBS-challenged mice. Moreover, *F. prausnitzii* and its SN restored these levels to normal. However, it is possible that the abundance of SERT reflects the general status of the epithelium, and it is not necessarily a direct consequence of the actions of *F prausnitizii* or its SN. Indeed, the LYBHI medium may have an effect on SERT. This is not the first time that culture media (LYBHI or MRS) have been reported to show intrinsic anti-inflammatory properties *in vivo* and *in vitro* [25]; this is presumably due to some of the components of the medium, as previously observed with MRS culture medium [55].

## Conclusion

Chronic low-grade inflammation is common in patients with IBD in clinical remission as well as in IBS patients [15,28]. Patients with histological inflammation at baseline are at increased risk of clinical relapse, hospitalization, surgery, and colon cancer in observational longitudinal studies [15]. It is widely recognized that microbiota can impact on gut homeostasis. Our study proposes that *F. prausnitzii*, one of the major members of the gut microbiota, protects from gut dysfunction-related symptoms in animal models. In summary, we describe novel protective effects of both *F. prausnitzii* and its SN on chronic low-grade inflammation, in a murine model mimicking the relapsing low-grade inflammatory conditions present in some IBD patients under remission. This bacterium exerts different beneficial effects in terms of cytokine levels, gut permeability and serotonin pathway. Furthermore, our results suggest that the protective effect of *F. prausnitzii* and its SN may be the consequence, at least in part, of barrier enhancement through certain tight junction proteins. These results provide further evidence of the potential of *F. prausnitzii* as a novel probiotic for gut-dysfunction management. Future studies will need to be performed to validate the described beneficial effect in patients gut dysfunction and low grade inflammation. Also, it will be important to determine whether disease associated dysbiosis is corrected, or whether the effect is independent of a modulation of the gut microbiota.

## Methods

### Bacterial strains and growth conditions

*Faecalibacterium prausnitzii* strain A2-165 (DSMZ collection, Braunschweig, Germany) (DSM N° 17677) was grown in LYBHI medium (brain-heart infusion medium supplemented with 0.5% yeast extract) (Difco, Detroit, USA) supplemented with 1 mg/ml cellobiose (Sigma-Aldrich Chemie GmbH, Buchs, Switzerland), 1 mg/ml maltose (Sigma-Aldrich), and 0.5 mg/ml cysteine (Sigma-Aldrich) at 37°C in an anaerobic chamber as previously described [22]. The supernatant of *F. prausnitzii* cultures (SN) were recovered by centrifugation filtered through 0.45 μm pore-size filters (VWR, Haasrode, Belgium) and stored at −80 C until use.

### Animals

Male C57BL/6 mice (6–8 weeks old) (Janvier, Le Genest Saint Isle, France or Taconic mice New York, USA) were maintained either at the animal care facilities of the National Institute of Agricultural Research (IERP, INRA, Jouy-en-Josas, France) or at the animal care facilities of McMaster University (CAF, Hamilton, Ontario, Canada) under specific pathogen-free conditions. Mice were housed under standard conditions for a minimum of one week before experimentation. All experiments were performed in accordance with European Community rules and approved by the Animal Care Committee COMETHEA (Comité d’Ethique en Expérimentation Animale du Centre INRA de Jouy-en-Josas et AgroParisTech, Jouy en Josas, France) and the Canadian Council on Animal Care Guidelines (CCAC).

### Low grade-inflammation induction and *F. prausnitzii* administration

The experimental protocol is detailed in Figure 1A. Briefly, mice of approximately 20 g were fully anesthetized by intraperitoneal (*i.p.*) injection of 150 μl of 0.1% ketamine (Imalgene 1000, Merial, France) and 0.06% xylazine (Rompun, Alcyon, France) and a 3.5 catheter (French catheter, Solomon Scientific, France) attached to a tuberculin syringe was inserted into the colon. A dose of 100 mg/Kg of DNBS solution (ICN, Biomedical Inc.) in 30% ethanol (EtOH) was then injected intrarectally (*i.r.*). Control mice (without colitis) received only 30% EtOH. Ten days after the DNBS acute period (the three days following DNBS administration during which the effects are greatest), 200 μl of 1X10^9^ CFU of bacteria in PBS, or PBS alone, or culture supernatant (SN) were administered intragastrically, daily for 10 days (gavage period). The DNBS administration was repeated 21 days after the first DNBS injection (recovery period) with a second injection of 50 mg/Kg of DNBS solution. Weight loss was monitored during 3 days following the second DNBS injection to assess possible clinical signs of distress.

To confirm the absence of overt inflammation, macroscopic and histological scores as well as myeloperoxidase (MPO) activity were determined as previously described [25].

### Lipocalin-2 (Lcn-2) levels

Before the mice were sacrificed, blood samples were obtained from the retro-orbital venous plexus, and centrifuged for 10 min at 12,000 rpm and 4°C. Clear supernatants (serum) were collected and stored at −20°C until analysis. Serum samples were diluted in the kit-recommended diluent (1% BSA in PBS), and Lcn-2 was assayed using Duoset murine Lcn-2 ELISA kit (R&D Systems, Minneapolis, MN) [36].

### Cytokine assays

One centimeter samples of distal colon were recovered and homogenized in an appropriate volume (to give 50 mg/ml) of Tris–HCl buffer containing protease inhibitors (Sigma-Aldrich) in a Tissue Lyser (Qiagen). The samples were centrifuged for 20 min and the supernatants collected and frozen at −80°C until analysis. Blood samples were obtained from the retro-orbital venous plexus before the mice were killed and centrifuged, and the sera stored at −80°C until analysis. IL-6, IL-10, IFN-γ, TNF-α, IL-5, IL-2, IL-22, IL-1α, IL-13, IL-17, IL-4, IL-27 and IL-12p70 were assayed with a cytometric bead array system (Mouse Th1/Th2/Th17/Th22 13plex Flowcytomix) (eBioscience, San Diego, USA).

### Intestinal permeability *in vitro* and *in vivo*

Three days after the final DNBS challenge, intestinal permeability *in vitro* was assessed by the Ussing chamber technique as previously described (World Precision Instruments, Sarasota, FL, USA) [56,57]. Two sections of colon from each mouse were assessed. Tissue conductance (the passive permeability to ions) and mucosal-to-serosal flux of the paracellular probe ^51^Cr-EDTA were determined to assess barrier function and paracellular permeability. ^51^Cr-EDTA flux was calculated as the average over a 2-h period and is expressed as %hot/cm^2^/hr.

Permeability *in vivo* was assessed using fluoroscein-conjugated dextran (FITC-dextran 3000–5000 Da, Sigma-Aldrich) as previously described [58]. Briefly, three days after the second DNBS injection, 0.6 mg/g body weight of FITC-dextran was administered to mice by oral gavage and 3.5 h later, blood samples were obtained from the retro-orbital venous plexus. Plasma FITC levels were determined by fluorometry using a microplate reader (Tecan, Lyon, France).

### Apical junctional analysis by quantitative real-time PCR (qPCR) and immunostaining

Total RNA was isolated from 20–30 mg samples of colon with an RNeasy Mini Kit (Qiagen). Column DNAse treatment (Qiagen) was used to eliminate potential DNA contamination. RNA quantity and quality were checked with a NanoDrop aparatus (Thermo Scientific) and by agarose gel electrophoresis. Only samples with intact RNA were used for subsequent cDNA synthesis with iScript reverse transcriptase (Bio-Rad): 500 μg of the total RNA preparation was used for each sample. Quantitative real-time PCR (qPCR) was performed with diluted cDNA (10x) in triplicate and with an iQ5 Real-Time Detection System (Bio-Rad). The reaction mix consisted of Sofast Evagreen Supermix (Bio-Rad), primers at 0.5 μM (Additional file 2: Table S1), and 2 μL of diluted cDNA. Values are expressed as relative fold differences normalized to a housekeeping gene, *Gapdh,* by the 2^-ΔΔCT^method. All procedures were performed according to the manufacturers’ instructions.

Protein expression of apical junctional proteins was evaluated also using immunofluorescence. Colon samples were embedded in Tissue-Tek OCT (Sakura, Torrance, CA). Frozen sections were then cut (5 μm), fixed with 3% paraformaldehyde (PFA) for 15 minutes at 20°C, and blocked with phosphate-buffered saline (PBS)/bovine serum albumin at 2% for 1 hour. Samples were immune-stained overnight with E-cadherin antibody (1:1000 dilution, BD Pharmaceutical), Occludin (1:200, Invitrogen), Claudin 4 (1:200, Invitrogen), F11r (1:100, R&D) and 1 hour with appropriate secondary antibody (1:250 dilution, Molecular Probes). Representative pictures from each animal were taken with the same exposure time.

### Colonic serotonin assays

One centimeter-long sections of colon were recovered and homogenized (50 mg/ml) in PBS buffer containing protease inhibitors and Serotonin Stabilizer (LDN, Nordhon, Germany) in a Tissue Lyser (Qiagen). The colon contents were recovered by washing with 1 ml of PBS buffer containing protease inhibitors and Serotonin Stabilizer (LDN) and centrifuged; the supernatant stored at −80°C until analysis. Serotonin concentrations were determined using a Serotonin Research ELISA (LDN) according to the manufacturer’s instructions.

### Serotonin transporter (SERT) expression in colon

Total RNA was extracted from 1 cm-long sections of colon homogenized in a TissueLyser, by the guanidinium thiocyanate method [59]. RNA concentration and purity were determined using a Nanodrop ND-1000 apparatus (Thermo Scientific) and the RNA Integrity Number (RIN) was checked using an Agilent 2100 bioanalyzer (ICE platform, INRA, Jouy-en-Josas, France) and an RNA 6000 Nano LabChip® kit (Agilent technologies). Reverse transcription was performed with 0.1 to 5 μg of RNA of each sample using the High-Capacity cDNA Archive Kit (Applied Biosystems, France) according to the manufacturer’s instructions. The absence of PCR inhibition was confirmed for each cDNA sample with the TaqMan® Exogenous Internal Positive Control (Applied Biosytems, France). The cDNA products were analyzed in triplicate by RT-qPCR with an ABI PRISM 7000 Sequence Detection System and the 7000 system software version 1.2.3 (Applied Biosytems). Several genes were analyzed using TaqMan Gene Expression Assays (Applied Biosystems, France), including *Slc6a4* (Mm00439391_m1) and *gapdh* (Mm99999915_g1), used as a reference. The results obtained were normalized to the value for *gapdh* rRNA and compared with the mean target gene expression in the ETOH-PBS group as the calibrator sample. The following formula was used: fold change = 2^-ΔΔCt^, where ΔΔCt threshold cycle (Ct) equals (target Ct - reference Ct) of sample minus (target Ct - reference Ct) of the calibrator.

### Statistical analysis

GraphPad software (GraphPad Sofware, La Jolla, CA, USA) was used for statistical analysis. Results are presented as bar graphs or dot plots with means +/− SEM. Comparisons involved the non-parametric Kruskal-Wallis test was used, followed by a Dunn’s Multiple Comparison test. A *p* value of less than 0.05 was considered significant.

## Additional files

Additional file 1: Figure S1. Effect of *F. prausnitzii* on apical junction proteins in a DNBS-induced low-grade inflammation model. Sections of the distal colon were stained for Claudin-4, Fr11, occludin (green) and E-cadherin (red) expression. Nuclei (DAPI; blue). Original magnification X20. Representative images from control non-inflamed (EtOH-PBS), control inflamed (DNBS-PBS), bacteria-free culture medium (DNBSLYBHI), *F. prausnitzii* strain A2-165 (DNBS-Fprau), and *F. prausnitzii* A2-165 SN (DNBS-SN) groups. Additional file 2: Table S1. Primers used in this study.

## Abbreviations

AJ: Adherens junction proteins
DNBS: Dinitro-benzene sulfonic acid
EC: Enterochromaffin cells
EtOH: Ethanol
H&E: Hematoxylin and eosin
IBD: Inflammatory bowel disease
*i.p.*: Intraperitoneal
*i.r.*: Intrarectally
Lcn-2: Lipocalin 72
LYBHI: Brain-heart infusion medium supplemented with 0.5% yeast extract
MPO: Myeloperoxidase
PFA: Paraformaldehyde
qPCR: Quantitative real-time PCR
5-HT: Serotonin
SERT: Serotonin reuptake transporter
SN: Supernatant
TJ: Tight junction proteins
