# Supplementary figures and images for: Faecalibacterium prausnitzii prevents physiological damages in a chronic low-grade inflammation murine model
Source: BMC Microbiol. 2015 Mar 21;15:67. doi: 10.1186/s12866-015-0400-1 (PMC4391109; doi:10.1186/s12866-015-0400-1)

## Slide 1
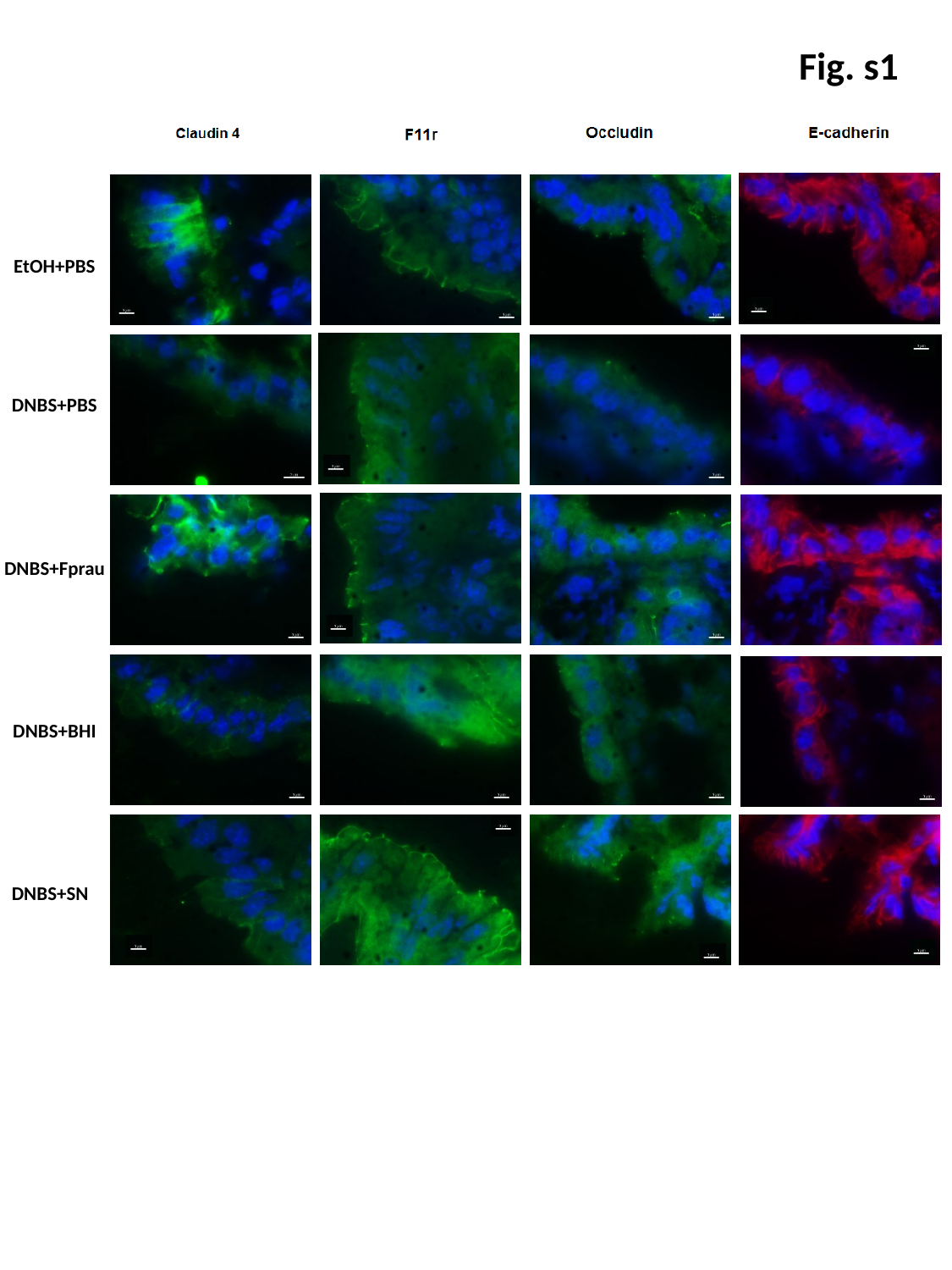

Fig. s1
EtOH+PBS
DNBS+PBS
DNBS+Fprau
DNBS+BHI
DNBS+SN

Supplement: Additional file 1: Figure S1. — Effect of F. prausnitzii on apical junction proteins in a DNBS-induced low-grade inflammation model. Sections of the distal colon were stained for Claudin-4, Fr11, occludin (green) and E-cadherin (red) expression. Nuclei (DAPI; blue). Original magnification X20. Representative images from control non-inflamed (EtOH-PBS), control inflamed (DNBS-PBS), bacteria-free culture medium (DNBSLYBHI), F. prausnitzii strain A2-165 (DNBS-Fprau), and F. prausnitzii A2-165 SN (DNBS-SN) groups. [file 12866_2015_400_MOESM1_ESM.ppt]
