# Supplementary material for: Faecalibacterium prausnitzii prevents physiological damages in a chronic low-grade inflammation murine model
Source: BMC Microbiol. 2015 Mar 21;15:67. doi: 10.1186/s12866-015-0400-1 (PMC4391109; doi:10.1186/s12866-015-0400-1)
Supplement: Additional file 2: Table S1. — Primers used in this study. [file 12866_2015_400_MOESM2_ESM.doc]

**Additional file 2: Table S1. Primers** used in this study

| **Gene** | **Sequence** | **T-Cº** | **Product Size (bp)** | **Efficiency** |
| --- | --- | --- | --- | --- |
| **Cell Surface Receptors** | | | | |
| Cldn1(NM_016674) | F: GGACTGTGGATGTCCTGCGTTT  R: GCCAATTACCATCAAGGCTCGG | 60 | 117 | 95% |
| Cldn2(NM_016675) | F: AGGACTTCCTGCTGACATCCAG  R: AATCCTGGCAGAACACGGTGCA | 59 | 119 | 101% |
| Cldn3(NM_009902) | F: TCATCGTGGTGTCCATCCTGCT  R: AGAGCCGCCAACAGGAAAAGCA | 59 | 142 | 99% |
| Cldn4(NM_009903) | F: CGAGCCCTTATGGTCATCAGCA  R: ATGCTTGCCACGATGAACACGG | 59 | 149 | 98% |
| Cldn5 (NM_013805) | F: TGACTGCCTTCCTGGACCACAA  R:CATACACCTTGCACTGCATGTGC | 59 | 108 | 104% |
| Cldn15 (NM_021719) | F: AACTGCTGGGACTTTCCGTCCA  R: TGGAGAGATCCATGTTGCCCAC | 59 | 154 | 102% |
| Ocln(NM_008756) | F: TGGCAAGCGATCATACCCAGAG  R: CTGCCTGAAGTCATCCACACTC | 60 | 103 | 99% |
| **Cytoplasmic Cell Receptor** | | | | |
| Tjp1 (ZO-1) (NM_001163574) | F: GTTGGTACGGTGCCCTGAAAGA  R: GCTGACAGGTAGGACAGACGAT | 60 | 133 | 97% |
| **Cell Adhesion Molecule** | | | | |
| Cdh1 (E-cadherin) (NM_009864) | F: GCACATATGTAGCTCTCATC  R: CCTTCACAGTCACACACATG | 59 | 394 | 98% |
| **Junctional Adhesion Molecule** | | | | |
| F11r (JAM-A) (NM_172647) | F: CACCTACTCTGGCTTCTCCTCT  R: TGCCACTGGATGAGAAGGTGAC | 59 | 131 | 105% |
| **Housekeeping Gene** | | | | |
| Gapdh  (NM_008084 ) | F: CCATGGAGAAGGCTGGGG  R: CAAAGTTGTCATGGATGACC | 56-60 | 195 | 101% |
